# Macrophage-Mediated Glial Cell Elimination in the Postnatal Mouse Cochlea

**DOI:** 10.3389/fnmol.2017.00407

**Published:** 2017-12-11

**Authors:** LaShardai N. Brown, Yazhi Xing, Kenyaria V. Noble, Jeremy L. Barth, Clarisse H. Panganiban, Nancy M. Smythe, Mary C. Bridges, Juhong Zhu, Hainan Lang

**Affiliations:** ^1^Department of Pathology and Laboratory Medicine, Medical University of South Carolina, Charleston, SC, United States; ^2^Department of Otorhinolaryngology, Shanghai Jiao Tong University Affiliated Sixth People’s Hospital, Otolaryngology Institute of Shanghai Jiao Tong University, Shanghai, China; ^3^Department of Regenerative Medicine and Cell Biology, Medical University of South Carolina, Charleston, SC, United States

**Keywords:** auditory nerve, cochlea, hearing onset, macrophage, glial cells, CD11b

## Abstract

Hearing relies on the transmission of auditory information from sensory hair cells (HCs) to the brain through the auditory nerve. This relay of information requires HCs to be innervated by spiral ganglion neurons (SGNs) in an exclusive manner and SGNs to be ensheathed by myelinating and non-myelinating glial cells. In the developing auditory nerve, mistargeted SGN axons are retracted or pruned and excessive cells are cleared in a process referred to as nerve refinement. Whether auditory glial cells are eliminated during auditory nerve refinement is unknown. Using early postnatal mice of either sex, we show that glial cell numbers decrease after the first postnatal week, corresponding temporally with nerve refinement in the developing auditory nerve. Additionally, expression of immune-related genes was upregulated and macrophage numbers increase in a manner coinciding with the reduction of glial cell numbers. Transient depletion of macrophages during early auditory nerve development, using transgenic CD11b^DTR/EGFP^ mice, resulted in the appearance of excessive glial cells. Macrophage depletion caused abnormalities in myelin formation and transient edema of the stria vascularis. Macrophage-depleted mice also showed auditory function impairment that partially recovered in adulthood. These findings demonstrate that macrophages contribute to the regulation of glial cell number during postnatal development of the cochlea and that glial cells play a critical role in hearing onset and auditory nerve maturation.

## Introduction

The auditory nerve is the conduit for transmission of sound in the mammalian ear. Axonal projections of bipolar spiral ganglion neurons (SGNs) connect sensory hair cells (HCs) to central auditory processes in the brain. Two subpopulations of SGNs exist: type I, which account for 90%–95% of total SGNs, and type II, which comprise the remaining 5%–10% of SGNs. The cell bodies of both neuron types are housed within Rosenthal’s canal (RC) and extend peripheral axons through the osseous spiral lamina (OSL) to form connections with HCs in the organ of Corti (OC). Type I SGNs extend a single axon to a single inner hair cell (IHC) and type II SGNs innervate multiple outer hair cells (OHC) with numerous axonal projections (Marrs and Spirou, 2012). SGNs begin to innervate cochlear HCs in a tonotopic pattern as early as embryonic day (E) 15.5 (Koundakjian et al., 2007). At this time, multiple type I SGNs extend axons past the IHC region, forming erroneous connections with OHCs. Similarly, type II SGN axons form mistargeted connections with IHCs (Echteler and Nofsinger, 2000; Huang et al., 2007; Druckenbrod and Goodrich, 2015). Between E17.5 and the first postnatal week in rodents, excessive type I and II axons are eliminated by retraction or pruning to produce mature innervation patterns (Echteler, 1992; Echteler and Nofsinger, 2000; Echteler et al., 2005; Huang et al., 2007; Yu et al., 2013). This refinement process occurs prior to hearing onset, which commences between P12–P14 (Song et al., 2006).

SGNs and auditory nerve fibers are wrapped by neural crest-derived auditory glial cells (Hansen et al., 2001; Breuskin et al., 2010). Type I SGN somas are covered by myelinating satellite cells and their axons are ensheathed by myelinating Schwann cells. Conversely, type II SGNs are enveloped by non-myelinating satellite and Schwann cells. Recent studies have demonstrated that migration, maturity and survival of SGNs is largely dependent on surrounding glial cells (Hansen et al., 2001; Stankovic et al., 2004; Jeon et al., 2011; Lang et al., 2011, 2015). Additionally, conditional deletion of Sox10, a transcription factor regulating glial cell differentiation, results in reduced glial numbers, abnormal SGN distribution, decreased SGN migration, and the appearance of nerve fibers that overshoot the OC and extend into the cochlear lateral wall (LW), revealing the important role glial cells play in proper SGN innervation (Mao et al., 2014). However, it is still unknown how glial cell numbers are regulated as the auditory nerve matures.

Immune cells such as macrophages and microglia contribute to nerve refinement in the central nervous system (Paolicelli et al., 2011; Schafer et al., 2012; Cunningham et al., 2013; Miron et al., 2013). Macrophages, monocyte-derived phagocytes, are members of the innate immune system and respond to various cytokine/chemokine signals to remove pathogens and apoptotic cells (Tang et al., 2006; Guipponi et al., 2007; Akil et al., 2015). Macrophages have also been detected in the adult cochlea following cochlear insult (Hirose et al., 2005; Lang et al., 2006; Sato et al., 2010; Kaur et al., 2015). Damaged SGNs, auditory glial cells and fibrocytes secrete pro-inflammatory signals that promote the infiltration of macrophages (Bas et al., 2015; Fuentes-Santamaría et al., 2017). Recent studies have shown that immune-related gene expression increases in the developing auditory nerve (Lu et al., 2011; Bank et al., 2012; Calton et al., 2014). However, the role of macrophages in auditory development has not been characterized.

Here, we report that macrophages are implicated in the clearance of glial cells during development. This was supported by a dynamic reduction in glial cell number and accompanying evidence of macrophages engulfing glial cells and degenerative axons. Depletion of activated macrophages in mice caused glial cell alterations, myelin dysfunction and transient edema of the stria vascularis. Additionally, macrophage-depleted mice exhibited transient auditory function impairment. Collectively, these findings reveal that macrophages play a novel role in glial cell maintenance during auditory nerve maturation.

## Materials and Methods

### Animals

CBA/CaJ and CD11b^DTR/EGFP^ mice were bred in-house in a low-noise environment at the Animal Research Facility of the Medical University of South Carolina (MUSC; Charleston, SC, USA). All aspects of animal research were conducted in accordance with the guidelines of the Institutional Animal Care and Use Committee of MUSC and the Guide for the Care and Use of Laboratory Animals of the National Institutes of Health. The protocols (2290 and 2845) were approved by the IACUC of the MUSC. The original adult CBA/CaJ and CD11b^DTR/EGFP^ breeding pairs were purchased from The Jackson Laboratory (Bar Harbor, ME, USA). All mice received food and water *ad libitum*, and were maintained on a 12-h light/dark cycle. For mouse cochlear collections, the day of birth was referred to as postnatal day 0 (P0). Since sex is grossly indistinguishable until P14 in mice, pups of either sex were used in this study. Young adult mice of either sex were also used in this study.

### Diphtheria Toxin Administration

P4–5 CD11b^DTR/EGFP^ mice received intraperitoneal injections (IP) of diphtheria toxin (DTX; 20–25 ng/g of body weight; Sigma-Aldrich) reconstituted in double distilled H_2_O (ddH_2_O). It has been reported that vehicle-treated CD11b^DTR/EGFP^ mice and DTX-treated non-transgenic mice of the same background do not demonstrate auditory functional differences (Zhang et al., 2012). To assure age-matching between mice, CD11b^DTR/EGFP^ littermates injected with vehicle solutions and were used as controls.

### BrdU Postnatal Injections

Bromodeoxyuridine (BrdU, 100 mg/kg, Sigma-Aldrich) was administered to neonatal CBA/CaJ mice by IP injection daily for postnatal days P0–P3. BrdU incorporation analysis was measured at P3, P7, P14 and P21 (details in the “Immunohistochemistry” section below).

### Immunohistochemistry

Cochleae were fixed with 4% paraformaldehyde solution for 1–2 h at room temperature (RT), decalcified with 0.12 M ethylenediamine tetra acetic acid (EDTA) at RT with stirring, and cryopreserved with 30% sucrose for 30 min. The tissues were then embedded in Tissue-Tek OCT Compound (VWR, Radnor, PA, USA) and stained as whole-mount preparations or sectioned at 10 μm thickness. The primary antibodies used in this study include: rabbit anti-Iba1 (Wako Chemicals USA Inc., Richmond, VA, USA (1:250)), rat anti-CD11b (Serotec, Oxford, UK (1:150)), mouse anti-CD68 (Abcam, Cambridge, MA, USA (1:100)), rabbit anti-F4/80 (Abcam (1:200)), mouse anti-Neurofilament 200 (Sigma-Aldrich (1:200)), rabbit anti-Myosin VIIa (Proteus BioSciences, Ramona, CA, USA (1:200)), mouse anti-C terminal binding protein 2 (BD Biosciences, San Jose, CA, USA (1:200)) and goat anti-Sox10 (Santa Cruz Biotechnology, Santa Cruz, CA, USA (1:80)). Secondary antibodies were biotinylated and binding was detected by labeling with fluorescein (FITC)-conjugated avidin D, Texas Red-conjugated avidin D (Vector Labs, Burlingame, CA, USA) or Alexa Fluor Dyes (ThermoFisher Scientific, Waltham, MA, USA). Nuclei were counterstained with propidium iodide (PI) or 4′,6-diamidino-2-phenylindole (DAPI).

The mouse monoclonal anti-BrdU was produced by clone BU33 and raised against BrdU incorporated into DNA, or coupled to a protein carrier. It recognized proliferative cells in the nuclei of frozen sections of animals treated with an *in vivo* administration of BrdU. In addition to the immunohistochemistry steps described above, BrdU-labeled sections were treated with two moles of hydrogen chloride for 30 min and 0.1 M of sodium borate buffer for 5 min prior to biotinylation.

Sections were examined on a Zeiss LSM5 Pascal (Carl Zeiss Inc., Jena, DE, Germany) confocal microscope, a Zeiss LSM 880 NLO or Leica TCS SP5 (Leica Microsystems, Allendale, NJ, USA) confocal microscope. FITC and Texas Red signals were detected by excitation with the 488 nm and 543 nm lines, respectively. Images were scanned at image scales of 225.0 μm (x) × 225.0 μm (y), 144.72 μm (x) × 144.72 (y) and 450.0 μm (x) × 450.0 μm (y). Captured images were processed using Zen 2012 Blue acquisition software (Zeiss Inc.), Leica Application Suite X software (Version 3.0.2.16120) and Adobe Photoshop CS6 (Adobe Systems Inc., San Jose, CA, USA).

### Histology Quantification

Quantitative analysis of macrophages, glial cells and proliferative cell numbers were determined using AxioVision 4.8 (Carl Zeiss, Inc.) software. Regions of interests were determined by outlining intact RC and OSL, defined as boundaries from the habenular opening to a proximal site near the spiral ganglia, areas using the software outline tool. Similar tonotopic region sizes were examined between different cochlear samples. Within each region of interest, total cell numbers were determined by counting PI or DAPI counterstained cell nuclei using the measurement tool. Measurements of macrophages, glial cells, neurons and proliferative cells were determined by counting cells immunolabeled for Iba1^+^, Sox10^+^, NF200^+^ or BrdU^+^, respectively, in each region of interest. At least three slides from each ear from each postnatal age were used for data collection and processed using statistical analysis described below.

### Hair Cell and Synapse Quantification

Whole mount preparations of cochleae from P7 and 1 month DTX-treated and control CD11b^DTR/EGFP^ mice were stained with Myosin VIIa to identify IHCs and OHCs. HC numbers were counted manually using whole mount preparations from 1 month DTX-treated and control CD11b^DTR/EGFP^ mice (3 animals per group). Ribbon synapses under IHC were immunostained with CtBP2. CtBP2^+^ ribbons were measured manually from at least 10 IHCs in the apex, middle or base (3 animals per group). Confocal All images were taken with a Zeiss LSM 880 NLO using a 63× oil-immersion lens and acquired at 0.25 μm step size in the Z-axis in non-overlapping regions. Maximum projection images from confocal z-stacks were acquired with the same parameters described above. Care was taken to minimize pixel saturation while imaging each z-stack.

### Tissue Collection and Total RNA Isolation

Postnatal CBA/CaJ mice were euthanized and their cochleae were promptly collected. Microdissection was performed to remove the outer bony cochlear shell, cochlear LW and the majority of the sensory epithelium, preserving the modiolus portion of the cochlea. For RNA isolations, the left and right ear cochlea preparations from a single mouse were pooled for individual samples. Total RNA was purified from cochlea preparations using the miRNeasy Mini Kit (Qiagen Inc., Germantown, MD, USA) according to the manufacturer’s instructions.

### Microarray Data Analysis

A microarray dataset of mouse auditory nerve development from our group (NCBI Gene Expression Omnibus accession GSE59417; Lang et al., 2015) was used for comparative analysis. The dataset contains expression data for auditory nerve samples collected at P0, 3, 7, 10, 14 and 21 analyzed by Mouse 430 2.0 GeneChip (Affymetrix, Santa Clara, CA, USA). Raw hybridization data was normalized independently by both Robust Multi-array Average and MicroArray Suite 5.0 algorithms using Expression Console Software (Affymetrix). Differential expression was defined as absolute signal log ratio >0.5, ≥50% present gene detection scores and *p* ≤ 0.05 (Student’s *t*-test, unpaired) for P7 vs. P0 (identifying 2391 probe sets). False discovery rate was estimated at 1.7% based on iterative comparisons involving randomized sample group assignments. Among the 2391 probe sets, 1878 analysis-ready genes were found. Functional analysis and canonical function identification was assessed with Ingenuity Pathway Analysis Software version (IPA^®^; QIAGEN, Redwood City, CA, USA).

### Quantitative PCR (qPCR)

Reverse transcription of total RNA was performed with the QuantiTect Reverse Transcription Kit (Qiagen) according to the manufacturer’s protocol. Briefly, genomic DNA was eliminated from RNA samples by incubation with gDNA Wipeout Buffer for 2 min at 42°C. Reverse transcription was performed with 50 ng of total RNA and Quantiscript Reverse Transcriptase in 20 μl reaction volumes incubated at 42°C for 15 min and 95°C for 3 min. The following gene-specific amplification reagents were purchased from Qiagen: H2D1 (QT01657761), H2K1 (QT01743700) C1qa (QT01057770), C3a receptor (C3ar; QT00251216), Ppfibp2 (QT00098161), and Runx1 (QT00100380). Quantitative PCR (qPCR) reactions were performed with the QuantiFast SYBR Green PCR Kit (Qiagen) using 1 μl of cDNA and a LightCycler 480 (Roche Diagnostics, IN, USA). Negative controls included reactions lacking cDNA template and reverse transcriptase. All reactions were performed in technical triplicate. Cycling parameters for PCR were: 50°C for 2 min, activation at 95°C for 5 min, and 40 cycles of 95°C for 10 s and 60°C for 30 s. Analysis of qPCR data was conducted with the aid of LightCycler 480 software (version1.5.0.39). Amplification efficiency of each target was determined according to the equation *E* = 10 (−1/S), where S is the slope of the standard curve generated from 10-fold serial dilutions of the DNA preparations. Relative expression levels were calculated using the ∆∆C_T_ method that involved calculated amplification efficiencies and then normalized to reference genes Hprt and 18*S*.

### Transmission Electron Microscopy

For animals aged P7, cochleae were collected and perfused through the oval window with 0.5 ml of a fixative mixture comprised of 4% paraformaldehyde and 2% glutaraldehyde in 0.1 M phosphate buffer, pH 7.4. For collection of animals aged P11 and P16, anesthetized animals were perfused via cardiac catheter with 10 ml of normal saline containing 0.1% sodium nitrite followed by 15 ml of a mixture of 4% paraformaldehyde and 2% glutaraldehyde in 0.1 M phosphate buffer, pH 7.4. After removing the stapes and opening the oval and round windows, 0.5 ml of the same fixative described above was perfused gently into the scala vestibuli through the oval window. Inner ears were dissected and immersed in fixative overnight at 4°C. Decalcification for P16 cochleae was completed by immersion in 40 ml of 120 mM solution of EDTA, pH 7.0, with gentle stirring at RT for 2–3 days with daily changes of the EDTA solution. Cochlear tissues were postfixed with 1% osmium tetroxide-1.5% ferrocyanide for 2 h in the dark, then dehydrated and embedded in Epon LX 112 resin. Semi-thin sections approximately 1 μm thick were cut and stained with toluidine blue. Ultrathin sections (70 nm thick) were stained with uranyl acetate and lead citrate and examined and imaged using a JEOL JEM-1010 transmission electron microscope (JEOL USA, Inc., Peabody, MA, USA).

### Auditory Brainstem Response Analysis

Auditory brainstem responses (ABRs) were measured as previously described (Lang et al., 2011, 2015). Mice were anesthetized by IP injection of xylazine (20 mg/kg) and ketamine (100 mg/kg) and placed in a sound-isolation room. The acoustic stimuli were generated using Tucker Davis Technologies equipment System III (Tucker-Davis Technologies, Gainsville, FL, USA) and a SigGen software package (Version 4.4.1). Calibration was completed using an IPC Microphone System (PCB Piezotronics Inc., Depew, NY, USA) in a probe tube clipped to the pinna. The signals were delivered into the animal ear canal through a 3–5 mm diameter plastic tube. ABRs were evoked at half octave frequencies from 4 kHz to 45.2 kHz with 5 ms duration tone pips with cos^2^ rise/fall times of 0.5 ms delivered at 31 times/s. Sound levels were reduced in 5 dB steps from 90 dB SPL to 10 dB SPL to determine thresholds. Distortion product otoacoustic emission testing was performed in mice under general anesthesia as previously described (Lang et al., 2010). Briefly, distortion product otoacoustic emissions (DPOAEs) were measured using a Tucker Davis Technologies equipment RZ6 system (Tucker Davis Technologies, Gainsville, FL, USA) and SigGen software using an ER-10B+ Lo Noise^TM^ Microphone System (Etymotic Research Inc., Grove Village, IL, USA). The acoustic assembly was tightly enclosed in the ear. Primary tones were swept from f2 = 5.6–45.2 kHz with f1/f2 ratio of 1.2. Physiological results of each mouse were analyzed for individual frequencies, and then averaged for each of these frequencies from 5.6 kHz to 45.2 kHz.

### Experimental Design and Statistical Analyses

Biological sample sizes were determined based on similar experiments from previous studies conducted by our laboratory (Jyothi et al., 2010; Lang et al., 2015, 2016) and other labs (Yu et al., 2013; Coate et al., 2015) with the goal of achieving stringent and accurate measures of difference, namely statistical power of ≥80% and significance level of ≤0.05. Statistical analyses were performed using GraphPad Prism 7 software (GraphPad Software Inc., La Jolla, CA, USA) for Windows. Quantitative data are expressed as mean ± SEM, unless otherwise specified. Sample size is indicated for each figure. Differences across groups with multiple comparisons were analyzed with One-way ANOVA with *post hoc* Bonferroni Multiple Comparison tests. Differences for single pairwise comparisons were analyzed using two-tailed, unpaired Student’s *t*-tests. Statistical significance was defined as a *p* value of ≤0.05; all significance values are indicated. Specific *p*-values and test statistics are reported in the table below. Only significant values are reported in the “Results” section.

## Results

### Glial Cell Numbers Decrease after the Second Postnatal Week

**Figure 1:**
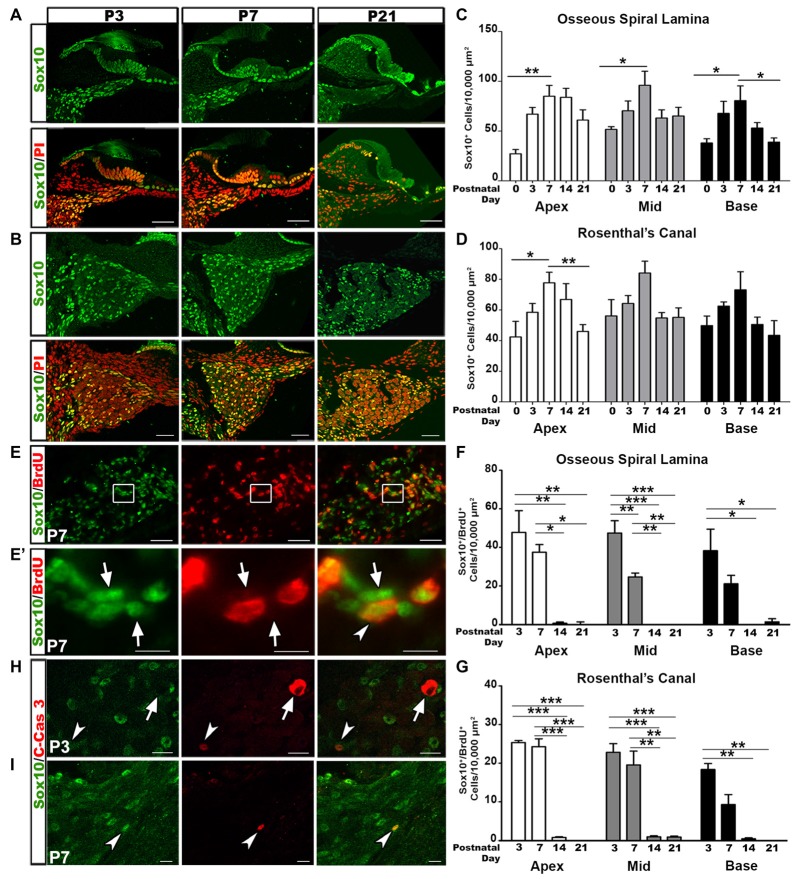
Increased glial cell numbers and proliferation correlate with auditory nerve refinement in postnatal mouse cochleae. **(A,B)** Confocal imaging of glial cell distribution in the postnatal cochlea. Sox10^+^ glia (green) and propidium iodide (PI; red) counterstained nuclei appeared in the osseous spiral lamina (OSL) **(A)** and Rosenthal’s canal (RC) **(B)** of P3, 7 and 21 mouse cochleae. Scale bars = 50 μm. **(C,D)** Quantitative analysis for Sox10^+^ cells in the OSL **(C)** and RC **(D)** in P0, 3, 7, 14 and 21 cochleae. Data bars represent mean density and error bars represent SEM. Two-tailed, unpaired Student’s *t*-tests were used to compare densities at P7 to the other time points for each turn (*p < 0.05; **p < 0.01; ***p < 0.001; *n* = 4–7 ears). The three cochlear turns are displayed: apical (white bars), middle (gray bars), and basal (black bars). **(E)** Dual-immunolabeling for Sox10 (green) and BrdU (red) identifies proliferative glial cells in the P7 RC. Cochlear samples were from mice that received a single intraperitoneal (IP) administration of BrdU at P0, P1 and P2. Scale Bars = 50 μm. **(E’)** Higher magnification of inset from **(E)** shows proliferative glia (arrows) and non-proliferative glia (arrowheads). Scale bar = 20 μm. **(F,G)** Quantitative analysis of Sox10^+^/BrdU^+^ cells in the OSL **(F)** and RC **(G)** for P3, 7, 14 and 21. One-way ANOVA followed by Bonferroni Multiple Comparison tests were used to compare densities between time points (*p < 0.05; **p < 0.01; *n* = 3–5 cochlear samples). Quantifications for the different cochlear turns are demonstrated: apical (white bars), middle (gray bars) and basal (black bars). Data bars represent mean density and error bars represent SEM. **(H,I)** Dual-immunolabeling for Sox10 (green) and C-Cas3 (cleaved-Caspase 3; red) reveals apoptotic glial cells (arrowheads) and apoptotic non-glial cells (arrow) in P3 (top) and P7 (bottom) cochleae. Scale Bars = 20 μm in **(H)**, Scale Bars = 20 μm in **(I)**. Apex, apical turn; Mid, middle turn; Base, basal turn.

SGNs undergo a rigorous course of refinement and retraction during postnatal development. Glial cells are in tight junction with SGNs. To determine changes in glial cell numbers during auditory nerve development we collected postnatal spiral ganglia at the ages of P0, P3, P7, P14 and P21 and calculated the density of Sox10^+^ cells. Sox10 transcription factor is a key developmental regulator expressed in both immature and differentiated glial cells of the auditory nerve (Britsch et al., 2001; Breuskin et al., 2010; Finzsch et al., 2010). Quantitative analyses of Sox10^+^ cells revealed that the highest density of glial cells in the OSL and RC occurred around P3–P7 (Figures 1A–D). After the first postnatal week, glial cell densities decreased. This temporal profile of glial cell numbers is in agreement with previous findings that the auditory nerve achieves a mature innervation pattern by the end of the first postnatal week in rodent models (Echteler, 1992; Huang et al., 2007; Coate et al., 2015; Druckenbrod and Goodrich, 2015).

To determine the proliferative ability of glial cells we utilized the BrdU assay. Cochlear samples were collected for analysis 3 days after BrdU injection. Double-labeling for BrdU and Sox10 revealed the presence of proliferative glial cells in the postnatal auditory nerve (Figures 1E,E′). Quantitative analysis of Sox10^+^/BrdU^+^ double-labeled cells revealed that the highest occurrence of glial cell proliferation occurred at P3–P7. Glial cell proliferation significantly decreased after the second postnatal week, after mature auditory nerve circuitry is established and SGNs are myelinated (Figures 1F,G).

The early phase of apoptosis is a rapid event necessary for the development of neural tissues and is often characterized by the cleavage of Caspase-3 (Jänicke et al., 1998; Hall, 1999; Porter and Jänicke, 1999). To determine if the temporal alterations in glial cells were due to apoptosis we examined cochlear samples from mice aged P3 through P14 for the presence of Sox10/cleaved-Caspase 3 positive cells. Very few cleaved-Caspase 3^+^ glial cells were identified in P3 and P7 cochleae (Figures 1H,I, arrowheads). Interestingly, one Sox10^-^ cell stained positive for cleaved-Caspase 3 in the P3 cochlea. The morphology of this apoptotic cell suggested it to be a SGN (Figure 1H, arrow), supporting previous research that found the highest incidences of SGN apoptosis in P4–6 rodent auditory nerves (Echteler et al., 2005). No cleaved-Caspase 3 positive cells were found in P14 cochlea (data not shown).

### Immune-Related and Axon Guidance Gene Expression Correlate with Auditory Nerve Maturation

**Figure 2:**
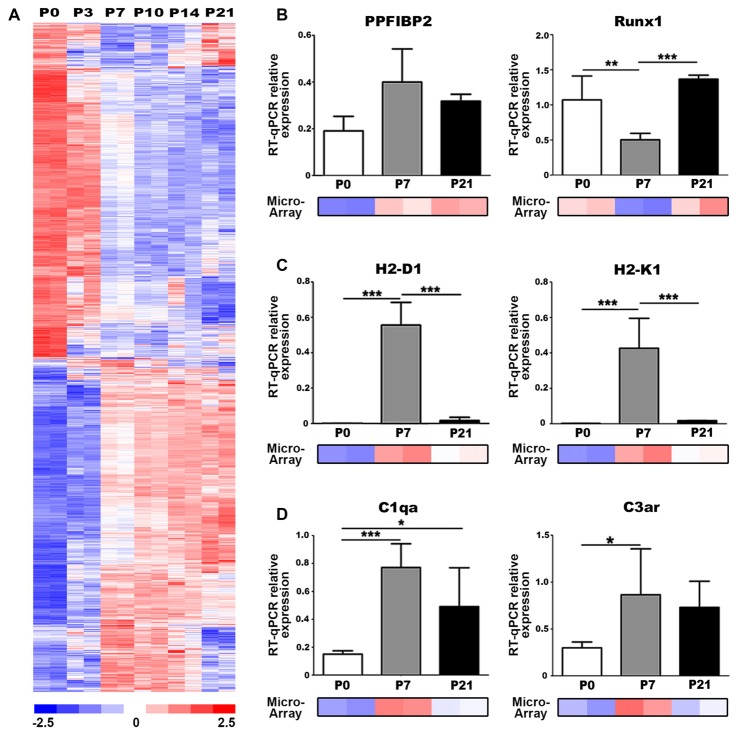
Genes associated with axonal guidance and immune responses are differentially expressed in the postnatal auditory nerve. **(A)** Expression profiles of genes differentially expressed between P0 and P7 in postnatal auditory nerves collected at P0, P3, P7, P10, P14 and P21. A total of 2391 probe sets, corresponding to 1878 genes, were identified comparing expression at P0 vs. P7 with absolute signal log ratio >0.5, ≥50% present gene detection scores and *p ≤* 0.05 (Student’s *t*-test). Colorimetric scaling (Z-standardization) for the heatmap is shown at the bottom. **(B–D)** Quantitative PCR (qPCR) validation for gene expression of **(B)** axonal guidance genes **(C)**, major histocompatibility class (MHC) II genes, and **(D)** complement pathway genes at P0 (black), P7 (gray) and P21 (white) is displayed. Microarray expression data at P0, P7 and P21 are demonstrated for each gene. One-way ANOVA with *post hoc* Bonferroni Multiple Comparison tests were used to compare gene expression between the postnatal days. Data bars represent average mean of experiments run in triplicate; error bars represent SEM; *p < 0.05; **p < 0.01, ***p < 0.001; *n* = 3.

We utilized our published microarray dataset of developing mouse auditory nerves (NCBI GEO:GSE59417; Lang et al., 2015) to identify molecular regulators and pathways involved in postnatal nerve development. We performed a comparison analysis of approximately 45,000 gene probe sets to identify genes that were differentially expressed between P0 and P7 (absolute signal log ratio >0.5; ≥50% present gene detection scores, Student’s unpaired *t*-test *p* ≤ 0.05). This analysis yielded 2391 probe sets with up- and downregulated expression values and an estimated false discovery rate of 1.7% (Figure 2A). Functional enrichment analysis was conducted using 1878 analysis-ready genes found among the 2391 probe sets. Pathway enrichment analysis detected 30 unique signaling pathways that were significantly enriched (Table 1), including Axonal Guidance Signaling, which is expected given that auditory nerve maturation occurs during the first postnatal week. Interestingly, several immune response pathways, including GADD45 Signaling and Complement System, were also significantly enriched, suggesting that immune-related activities are critical during auditory nerve development.

**Table 1:** Ingenuity pathway analysis of canonical pathways identified by differentially expressed genes.

Canonical pathway	*p*-value
Cell Cycle: G2/M DNA Damage Checkpoint Regulation	1.74E-04
Axonal Guidance Signaling	6.17E-04
GADD45 Signaling	3.02E-03
Cell Cycle Control of Chromosomal Replication	3.55E-03
Superpathway of Serine and Glycine Biosynthesis I	3.98E-03
dTMP *De Novo* Biosynthesis	4.90E-03
Melatonin Degradation II	4.90E-03
Estrogen-mediated S-phase Entry	5.50E-03
Osteoarthritis Pathway	8.51E-03
Adipogenesis pathway	9.77E-03
Serine Biosynthesis	1.15E-02
Glycine Degradation (Creatine Biosynthesis)	1.23E-02
Triacylglycerol Biosynthesis	1.41E-02
Hereditary Breast Cancer Signaling	1.66E-02
Phospholipases	2.04E-02
Pyrimidine Deoxyribonucleotides *De Novo* Biosynthesis I	2.29E-02
Antioxidant Action of Vitamin C	2.75E-02
Role of BRCA1 in DNA Damage Response	2.82E-02
Bladder Cancer Signaling	3.09E-02
Virus Entry via Endocytic Pathways	3.31E-02
Sphingomyelin Metabolism	3.39E-02
Sphingosine-1-phosphate Signaling	3.39E-02
Fatty Acid Activation	3.55E-02
Wnt/β-catenin Signaling	3.72E-02
Cell Cycle: G1/S Checkpoint Regulation	4.17E-02
Cholesterol Biosynthesis I	4.68E-02
Cholesterol Biosynthesis II (via 24,25-dihydrolanosterol)	4.68E-02
Cholesterol Biosynthesis III (via Desmosterol)	4.68E-02
ATM Signaling	4.79E-02
Complement System	4.90E-02

We performed qPCR to validate the temporal expression patterns of two key regulatory genes associated with axon guidance cues, *Ppfibp2* and *Runx1*, which were identified as differentially expressed in our analysis. *Ppfibp2* is a gene that encodes liprin beta-1 protein, a positive regulator of axonal extension and synapse formation (Dunah et al., 2005; Astigarraga et al., 2010). *Runx1* is a transcription factor that negatively regulates axonal projections and neuron maintenance (König et al., 2011; Yoshikawa et al., 2015). Although not significant, *Ppfibp2* showed a trend for increased gene expression from P0 to P7 and decreased by P21, in agreement with the microarray results (Figure 2B). We also found that *Runx1* expression significantly decreased at P7, when compared to P0 and P21 (Figure 2B), supporting the microarray results. Together, expression profiles of these genes indicate that P7 is a critical time point in auditory nerve maturation in the mouse cochlea.

The antigen presentation pathway was significantly enriched according to our functional analysis (Table 1). This pathway includes major histocompatibility class (MHC) class I genes, which signal for the targeting of nerves for elimination during synaptic pruning in spinal motorneurons (Thams et al., 2008). We performed qPCR to validate the expression of two MHC Class I genes, H2-D1 and H2-K1, which were identified as differentially expressed in our microarray analysis. Gene expression of H2-D1 and H2-K1 was upregulated in P7 auditory nerves, compared to P0 and P21 nerves, validating the expression values represented in the microarray dataset (Figure 2C). Complement signaling also mediates synaptic pruning and axon elimination in central and peripheral nerves (Schafer et al., 2012; Bialas and Stevens, 2013; Bahrini et al., 2015). We investigated the expression of two complement-related components identified as upregulated in our microarray dataset, complement component C1q, the molecular initiator of the classical complement pathway, and C3aR, a receptor for the central complement component for all complement pathways. qPCR confirmed that gene expression of C1q and C3aR was upregulated in the P0–P7 auditory nerve (Figure 2D).

### Macrophage Numbers Increase during Auditory Nerve Maturation

**Figure 3:**
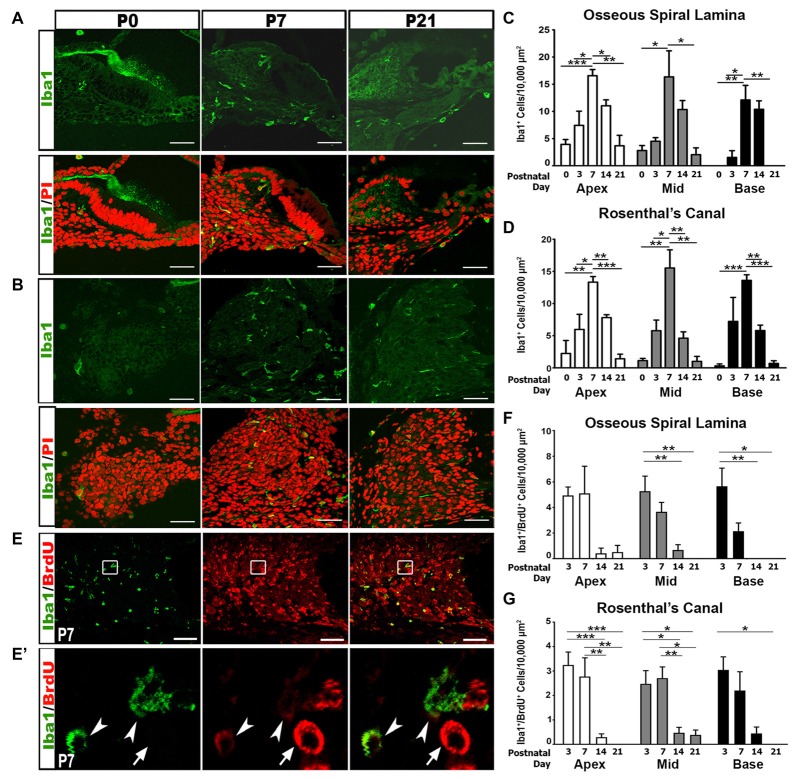
Iba1^+^ macrophage numbers and proliferation peak in the first postnatal week. **(A,B)** Confocal images show the temporal distribution of Iba1^+^ (green) macrophage cells in the OSL **(A)** and RC **(B)** of P0, P7 and P21 cochleae. Scale bars = 50 μm. **(C,D)** Quantitative analyses of Iba1^+^ macrophage densities in the OSL **(C)** and RC **(D)** of postnatal cochleae aged P0–P21 reveal an increase in macrophage numbers during nerve refinement. Quantifications for the different cochlear turns are displayed: apical (white bars), middle (gray bars) and basal (black bars). Data bars represent mean density and error bars represent SEM. Two-tailed, unpaired Student’s *t*-tests were used to compare Iba1^+^ macrophage densities at P7 to other postnatal time points in each turn (*p < 0.05; **p < 0.01; ***p < 0.001; *n* = 3–7 ears). **(E–G)** Confocal images show proliferating macrophages in the auditory nerve of a P7 cochlea. Macrophages are labeled with Iba1 (green) and proliferating cells are labeled with BrdU (red). Cochlear samples were from mice that received a single IP administration of BrdU at P0, P1 and P2. Scale bars = 50 μm. **(E′)** A high magnification image from enclosed area shown in **(E)** identifies proliferative macrophages (arrowheads) and a proliferative non-macrophage cell (arrow). **(F,G)** Quantitative analysis of BrdU/Iba1^+^ macrophage densities in the OSL **(F)** and RC **(G)** of postnatal cochleae aged P3–P21 reveals that most macrophage proliferation occurs during the first postnatal week (P3–7). One-way ANOVA with *post hoc* Bonferroni Multiple Comparison tests were used to compare densities between time points (*p < 0.05; **p < 0.01; ***p < 0.001 *n* = 3–7 ears). Quantifications for the different cochlear turns: apical (white bars), middle (gray bars) and basal (black bars). Data bars represent mean density and error bars represent SEM. Apex, apical turn; Mid, middle turn; Base, basal turn.

We further addressed the hypothesis that immune activities contribute to auditory nerve refinement by examining macrophage activity in the postnatal auditory nerve. Iba1, a highly conserved cytoplasmic protein, is specific to cells of monocytic lineage (Ohsawa et al., 2000; Sasaki et al., 2001). Analysis of Iba1^+^ cells revealed gradual increases in macrophage numbers in RC and the OSL from P0 to P3, reaching peak numbers at P7, and decreasing after P14 and P21 (Figures 3A–D). This increase in macrophage numbers coincides temporally with auditory nerve maturation and is subsequent to the glial cell increase.

To determine if the increase in macrophage density was due to the proliferation of resident macrophages, we injected BrdU into P0 pups and assessed macrophage proliferation. Dual labeling of Iba1^+^/BrdU^+^ cells demonstrated that the highest numbers of proliferative macrophages are seen during the first postnatal week (Figures 3E–G). Fewer proliferative macrophages were seen in the second and third postnatal weeks.

### Postnatal Macrophages Display Activated Phenotypes

**Figure 4:**
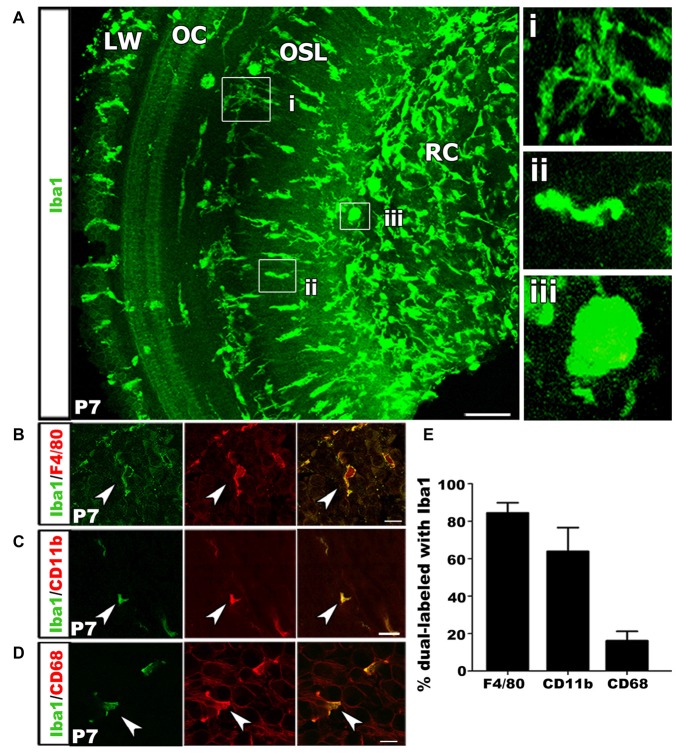
Macrophage activation occurs in the developing auditory nerve. **(A)** Confocal z-stack 3D rendering in mid-apical portion of a P7 mouse cochlea immunolabeled with Iba1 (green). Numerous Iba1^+^ macrophages are present in the OSL and RC with an ameboid shape, suggesting these Iba1^+^ cells are the activated macrophages. High magnification images from **(A)** shows a ramified macrophage **(i)** and an ameboid macrophage in the OSL **(ii)** and RC **(iii)**. LW, lateral wall; OC, organ of Corti; OSL, osseous spiral lamina; RC, Rosenthal’s canal. Scale bar = 50 μm. **(B)** Macrophages co-labeled with Iba1 (green) and F4/80 (red) antibodies (arrowhead). **(C)** An image of co-labeled macrophage cells with Iba1 (green) and CD11b (red), reveal macrophages at P7 are mature leukocytes (arrowhead). **(D)** Co-localization of Iba1 (green) and CD68 (red), a lysosomal glycoprotein marker, reveals phagocytic activity of cochlear macrophages (arrowhead). **(A–D)** Scale bars = 10 μm. **(E)** Bar graph depicting the percentage of Iba1^+^ cells that co-label with F4/80, CD11b and CD68. Percentages calculated from three to four mouse samples for each probe.

We performed immunohistochemical analysis of Iba1^+^ macrophages in postnatal tissue using cochlear whole mount preparation from P7 mice. This allowed visualization of entire cell morphologies. Macrophages exist in several distinct morphological states that are related to their functional roles. Ramified appearance indicates surveillance function, while rounded or ameboid shapes suggest phagocytic functions (Kaur et al., 2007; Parakalan et al., 2012). Iba1^+^ macrophages were present in multiple locations of the cochlea including RC, the OSL, along the outskirts of and within the OC and in the cochlear LW (Figure 4A). Investigation of macrophage morphology revealed that some macrophages were ramified (Figure 4Ai) while others were ameboid (Figures 4Aii,iii).

To further characterize the macrophage phenotype the postnatal auditory nerve, we probed for a panel of lysosomal and surface protein markers that are expressed by mature monocytes and during immune cell activation. We first performed immunostaining for F4/80, a marker for glycoprotein presented on the surface of mature monocytes (Austyn and Gordon, 1981; Hume et al., 1984). Over 80% of Iba1^+^ cells in the P7 cochlea stained positive for F4/80, confirming the differentiated phenotype and mature identity of the Iba1^+^ cells in the auditory nerve (Figures 4B,E). Next, we assessed the activation and phagocytic profile of the immune cells in P3–7 auditory nerves by probing for immunoreactivity of CD11b, a cell surface integrin indicative of macrophage/microglia activation, and CD68, a lysosomal protein expressed during debris degradation (Barros et al., 2013; Hickman et al., 2013; Bennett et al., 2016). The presence of Iba1^+^/CD11b^+^ and Iba1^+^/CD68^+^ cells suggested that a portion of macrophages engulfed particles and were actively degrading debris and cell fragments (Figures 4C–E).

### Macrophages Phagocytose Glial Cells and Axonal Fragments

**Figure 5:**
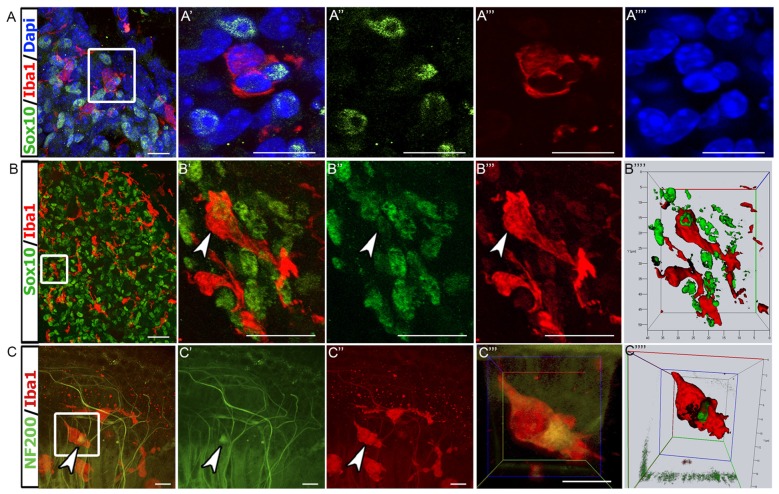
Macrophages engulf glial cells and the fragments of auditory nerve fibers. **(A–A″″)** Confocal images of Sox10^+^ glial cells (green) and Iba1^+^ macrophages (red) in the P7 auditory nerve. Macrophages are in close proximity of glial cells. 4′,6-diamidino-2-phenylindole (DAPI; blue) was used to counterstain cell nuclei. Panel **(A′)** shows enlarged image from enclosed area. **(A–A″″)** Scale bars = 10 μm. **(B–B″″)** Another confocal image of Sox10^+^ cells (green) and Iba1^+^ macrophages (red) from a thick section of P7 auditory nerve. Panel **(B″″)** shows stacking and 3D rendering of the enclosed area reveals an Iba1^+^ macrophage (red) engulfing a Sox10^+^ glial cell (green; arrowhead). **(B–B″′)** Scale bars = 15 μm. **(C–C″″)** Confocal imaging of a P7 whole-mount cochlear preparation stained with Iba1 (red) and NF200 (green). Panel **(C″″)** shows stacking and 3D rendering of enclosed area shows an Iba1^+^ macrophage phagocytosing a NF200^+^ nerve fiber fragment (arrowhead). **(C–C″′)** Scale bars = 50 μm.

To further investigate the physical interaction between auditory glial cells and macrophages, 3D confocal microscopy was employed. We chose to examine the auditory nerve at P7, when cochlear macrophage numbers were the highest. Dual immunostaining for Sox10^+^ glia and Iba1^+^ macrophages in RC revealed that macrophages were in close proximity to glial cells (Figures 5A–B″′). Upon closer examination, it was apparent that Sox10^+^ glial cell nuclei were engulfed within the cytoplasm of macrophages (Figure 5B′). Internalization of glia by macrophages was confirmed by maximum projection analysis and 3D reconstructions of sections stained for Sox10 and Iba1 (Figure 5B″″). We then examined macrophage-axon relationships in the P7 auditory nerve. We assessed neurofilament 200^+^ (NF200) nerve fibers that extend through the OSL to reach their HC targets. Dual immunostaining of Iba1 and NF200 revealed that macrophages directly contact SGN axons in P7 cochleae (Figures 5C–C″′). Maximum projection images and 3D reconstruction confirmed that macrophages phagocytose and digest axonal fragments (Figure 5C″″).

**Figure 6:**
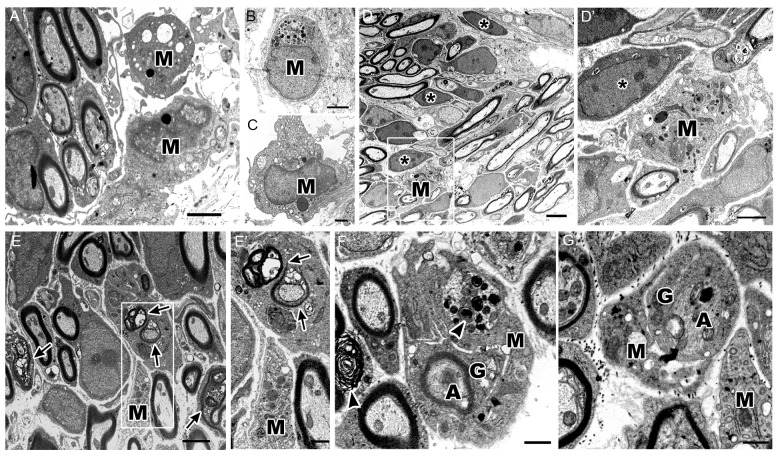
Ultrastructure of cochlea shows macrophages engulf degenerating axons and glial cells. **(A–C)** Micrographs of the P7 auditory nerve reveals cochlear macrophages (M) with activated morphologies including densely packed cytoplasm filled with multiple lysosomes, abundant rough endoplasmic reticulum, electron dense lipid bodies and phagoliposomes. **(A,B)** Scale bars = 2 μm, **(C)** Scale bar = 800 nm. **(D)** Electron micrograph shows myelinated axons in the OSL of a P7 mouse. Scale bar = 4 μm. Asterisks demonstrate supernumerary glial cells that are not juxtaposed to nerve fibers. **(D′)** Enclosed area highlights an activated macrophage (M) approaching a supernumerary glial cell (asterisk). Scale bar = 800 nm. **(E)** Micrograph of axons undergoing development-related degeneration (arrows). Scale bar = 2 μm. **(E′)** Enclosed area shows a macrophage (M) encroaching a glial cell with several degenerating axons within the cytoplasm (arrows). Scale bar = 800 nm. **(F)** The ultrastructure of the OSL area in another P7 mouse cochlea shows a macrophage (M) phagocytosing a glial cell (G) wrapping a myelinated, degenerative axon (A). Arrowheads highlight myelin blebs along degenerating axons and within the active macrophage. Scale bar = 800 nm. **(G)** Micrograph of a macrophage (M) engulfing a non-myelinating glial cell (G) and associated, degenerating axon (A). Scale bar = 600 nm. All images were composed from analysis of at least three mouse cochleae.

Using electron microscopy, we investigated the ultrastructure of different macrophage phenotypes in the auditory nerve of P7 cochleae. As expected, macrophages showed an active phenotype, including densely-packed cytoplasm filled with multiple lysosomes, abundant rough endoplasmic reticulum, electron-dense lipid bodies and phagoliposomes (Figures 6A–C). We also detected several glial cells that were not enclosing nerve fibers or neuron cell bodies (Figure 6D, asterisks). Interestingly, these free-floating, or supernumerary, glial cells were in close proximity to macrophages demonstrating activated morphologies (Figure 6D’). These observations correspond with the findings of glia-macrophage interactions identified by immunostaining and 3D reconstruction.

The ultrastructural analysis also detected SGN axons that were undergoing degeneration (Figure 6E, arrows). In some cases, a single glial cell ensheathed several degenerating axons (Figure 6E’). Active macrophages were seen encroaching on these degenerating SGN axons (Figure 6E’). There were also several macrophages that surrounded glia-wrapped degenerating axons (Figure 6F,G). Both myelinated and unmyelinated degenerating axons were engulfed by macrophages. These macrophages possessed myelin-like debris within their cytoplasm (Figure 6F, arrowheads), indicating that both glial cells and damaged or degenerative axons are phagocytosed by cochlear macrophages.

### Transient Depletion of Postnatal Macrophages Increases Glial Cell Numbers

**Figure 7:**
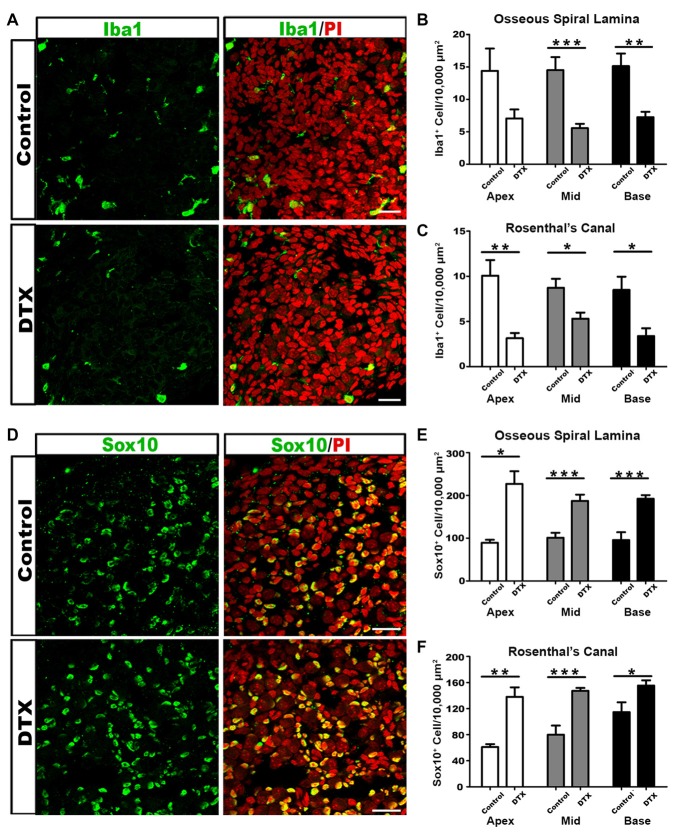
DTX treatment results in decreased macrophages and increased glial cells in the auditory nerve of postnatal CD11b^DTR/EGFP^ mice. **(A)** Confocal images of Iba1^+^ macrophages (green) and cell nuclei (PI-red) in middle turn RC of control (top panel) and DTX-treated (bottom panel) P7 cochleae. Scale bar = 25 μm. **(B,C)** Quantification of Iba1^+^ cells reveals fewer macrophages in DTX-treated OSL **(B)** and RC **(C)**, in comparison to controls. **(D)** Confocal images of Sox10^+^ glial cells in middle turn RC of control (top panel) and DTX-treated (bottom panel) P7 cochleae. Scale bars = 25 μm. **(E,F)** Quantification of Sox10^+^ glial cells reveals more glia in DTX-treated OSL **(E)** and RC **(F)**, in comparison to controls. Two-tailed, unpaired Student’s *t*-tests were used for statistical analyses (*p < 0.05; **p < 0.01; ***p < 0.001; *n* = 3–7 cochleae per treatment). Bars represent mean density and error bars represent SEM. DTX, diphtheria toxin; Apex, apical turn; Mid, middle turn; Base, basal turn.

To determine if macrophages are required to remove postnatal glial cells, we employed the CD11b^DTR/EGFP^ mouse model to transiently deplete active macrophages in the developing auditory nerve. Murine cells have low binding affinity for DTX. CD11b^DTR/EGFP^ mice express DTX receptors under the *CD11b* promoter. Administration of DTX to CD11b^DTR/EGFP^ mice results in a temporary depletion of only CD11b^+^ cells, with reductions lasting for 3–5 days (Cailhier et al., 2005; Stoneman et al., 2007; Ueno et al., 2013). To transiently deplete CD11b^+^ macrophages before the completion of auditory nerve maturation, DTX was administered to CD11b^DTR/EGFP^ pups at P4–5. Comparison of Iba1^+^ macrophage numbers in DTX- treated and control mice at P7 revealed significantly reduced macrophage numbers (Figures 7A–C). This corresponded with earlier results that demonstrated that a majority of Iba1^+^ cells also stained positive for CD11b (Figure 4C). Consequences on glial cell numbers were assessed by immunostaining with Sox10 in the OSL and RC (Figure 7D). We found that glial cells in the auditory nerve of DTX-treated mice were significantly increased compared to controls (Figures 7E,F). These results support the hypothesis that active macrophages regulate glial cell numbers during auditory nerve maturation.

**Figure 8:**
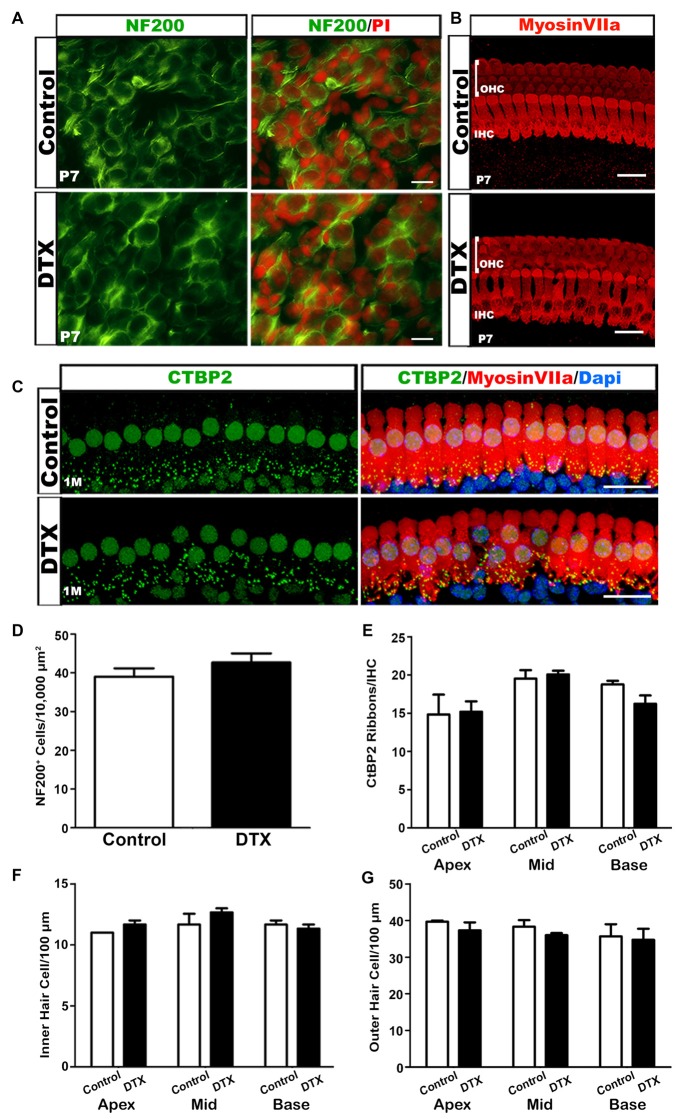
DTX-treated mice have relatively normal neuronal cells, hair cells (HCs) and afferent synapses. **(A)** NF200 (green) stained P7 cochlea show no difference between neurons of control and DTX-treated mice. PI (red) was used to counterstain nuclei. Scale bar = 25 μm. **(B)** Confocal imaging of Mysoin VIIa^+^ HCs in the OC of P7 control and DTX-treated mice. The intact three rows of outer hair cells (OHCs) and one row of inner hair cells (IHCs) were presented in both treatment conditions. Scale bar = 20 μm. **(C)** Immunostaining of Myosin VIIa (red) and CtBP2 (green) revealed no significant loss of afferent synapses under IHCs in 1 month old DTX-treated mice compared to controls. DAPI (blue) was used to counterstain cell nuclei. Scale bar = 20 μm. **(D)** Quantification of NF200^+^ neurons in P7 mice reveal no significant difference in spiral ganglion neuron (SGN) numbers in DTX-treated and control auditory nerves.**(E–G)** Quantitative analysis of Myosin VIIa and CtBP2 immunostaining in 1 month auditory nerves reveals no significant differences in IHC **(F)**, OHC **(G)** or ribbon synapse **(E)** numbers of DTX-treated and control mice.Two-tailed, unpaired Student’s *t*-tests were used for statistical analyses. No statistical significance was determined. *n* = 3 mice per group. Bars represent mean density and error bars represent SEM. Apex, apical turn; Mid, middle turn; Base, basal turn.

During the first postnatal week, specifically between P4–6, SGNs undergo a reduction in cell numbers due to an increase in apoptosis (Echteler et al., 2005). To ensure that DTX treatment did not alter the SGN population, we probed for NF200 in DTX-treated and control auditory nerves. There was no apparent difference in SGN distribution in DTX-treated and control auditory nerves (Figures 8A,D). This suggests that the mechanism of glial cell maintenance in macrophage-depleted nerves is not SGN-dependent. Whole mount preparations immunolabeled with MyosinVIIa were analyzed to investigate possible HC disruptions following DTX treatment. We found no detectable differences in the IHC or OHC numbers when comparing DTX-treated and control adult auditory nerves (Figures 8B,F,G). To investigate the effect of macrophage depletion on final afferent synapse distribution patterns, we immunostained whole-mount preparations of basilar membranes with anti-C-terminal binding protein (CtBP2) in adult CD11b^DTR/EGFP^ mice. Immunostaining of CtBP2 revealed no apparent difference in synapse distribution after DTX treatment (Figures 8C,E).

### Transient Depletion of Macrophages Results in Dysfunction of Myelinating Glial Cells

**Figure 9:**
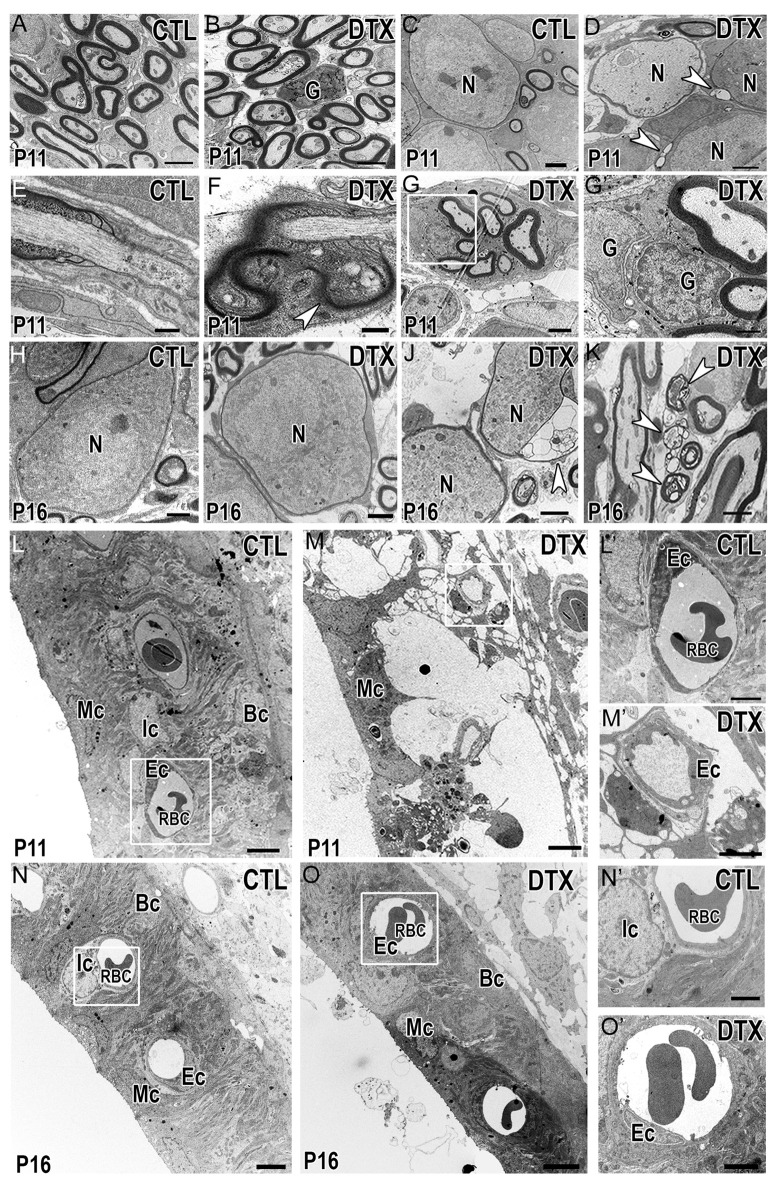
Depletion of macrophages results in transient myelin and LW-related abnormalities. **(A,B)** Electron micrographs demonstrate myelinated axons in control (CTL) **(A)** and DTX-treated (DTX) **(B)** auditory nerves of P11 CD11b^DTR/EGFP^ mice. A supernumerary glial cell can be found in the DTX-treated cochlea. Scale bars = 2 μm. **(C,D)** Micrograph illustrates myelinated neurons in control **(C)** and DTX-treated **(D)** auditory nerves. Arrowheads highlight vacuolations within the myelin of two neurons in the DTX-treated samples. Scale bars = 1 μm. **(E,F)** Micrographs of nodes of Ranvier in control **(E)** and DTX-treated **(F)** samples. Arrowhead shows thickened Schwann cell microvilli and myelin folding in the paranodal loops. Scale bars = 400 nm **(G)** Cluster of myelinated auditory nerve fibers in the OSL of P18 DTX-treated auditory nerves. Scale bars = 2 μm. **(G′)** High magnification of the enclosed area in **(G)** reveals supernumerary glial cells wrapping other glial cells. Scale bar = 800 nm. **(H–J)** Electron micrographs of neurons found in control **(H)** and DTX treated **(I)** auditory nerves of P16 CD11b^DTR/EGFP^ mice. **(J)** Arrowhead highlights blebbing in the myelin surround a neuron in the DTX-treated cochlea. Scale bars = 2 μm. **(K)** Glial cell containing several degenerative axons in the cytoplasm (arrowheads). Scale bar = 2 μm. **(L,M)** Micrographs of cochlear LWs from control **(L)** and DTX-treated **(M)** P11 CD11b^DTR/EGFP^ mice. Scale bars = 4 μm. **(L′,M′)** High magnification of the enclosed areas in L-M reveal blood vessel and endothelial cell morphology in control **(L′)** and DTX-treated **(M′)** cochleae. Scale bars = 2 μm. **(N,O)** Micrographs of cochlear LWs from control **(N)** and DTX-treated **(O)** P16 CD11b^DTR/EGFP^ mice reveals DTX-treated LWs are able to recover. Scale bars = 4 μm. **(N′,O′)** High magnifications of the enclosed areas in **(N,O)** reveal blood vessel and endothelial cells in control **(N’)** and DTX-treated **(O′)** cochleae. Scale bars = 2 μm. G, glial cell; N, spiral ganglion neuron; Mc, marginal cell; Ic, intermediate cell; Bc, basal cell; Ec, endothelial cell; RBC, red blood cell.

The production and compaction of myelin around SGN cell bodies and axons is a main function of auditory glial cells. Since myelin becomes more compact and mature between P10-P12 in rodent cochleae (Wang et al., 2013) we investigated the ultrastructure of the auditory nerves in P11 CD11b^DTR/EGFP^ mice. As expected, myelinated axons were seen in both control and DTX-treated auditory nerves (Figures 9A,B). DTX-treated nerves contained supernumerary glial cells that were not associated with nerve fibers or neuron cell bodies (Figure 9B). Myelinated SGNs were also seen in both control and DTX-treated nerves (Figures 9C,D); however, the myelin sheath surrounding SGNs of DTX-treated nerves showed modest alterations, including vacuolation and alveolated interruptions (Figure 9D, arrowheads). Nodes of Ranvier of DTX-treated nerves also demonstrated abnormalities, evidenced by robust myelin blebbing and folding in the paranodal regions (Figures 9E,F). Additionally, a group of myelinated axons were ensheathed by several Schwann cells (Figure 9G). High magnification revealed distinct cell boundaries were between the Schwann cells, indicating that supernumerary glial cells intermingle with and enwrap other myelinating Schwann cells (Figure 9G’). We investigated the auditory nerves of CD11b^DTR/EGFP^ mice at P16, a time after myelination is complete and after hearing onset. In P16 DTX-treated nerves, myelin appeared to recover and was comparable to that of control auditory nerves (Figures 9H,I). However, there were occasional cases of blebbing in the myelin surrounding neurons in DTX-treated nerves (Figure 9J). Additionally, few glial cells appeared to enwrap degenerating axons (Figure 9K).

CD11b^+^ cells have been found to reside in the cochlear LW of the adult cochlea. These cells, termed perivascular-resident macrophage-like melanocytes, remain tightly bound to LW blood vessels to control permeability within the intrastrial space of the cochlear LW (Zhang et al., 2012, 2013). We investigated the ultrastructure of the cochlear LW to determine if DTX-treatment altered LW cells (Figures 9L,M). Although marginal cells were maintained, losses of the interdigital processes of both marginal cells and intermediate cells with enlarged intrastrial space appeared in stria vascularis, indicating edema of the cochlear LW of P11 DTX-treated cochleae (Figure 9M). Endothelial cells surrounding blood vessels in DTX-treated LWs also showed ruffling in their cell membranes (Figure 9M’). This was not seen in endothelial cells of P11 control cochleae (Figure 9L’). Interestingly, examination of P16 DTX-treated and control cochleae revealed that edema of the stria vascularis was no longer present and endothelial cells surrounding LW blood vessels were comparable to those present in control cochleae (Figures 9N–O’). Our analysis of macrophage-depleted cochleae agrees with recent studies that reported recovery/self-repairing capability of the cochlear LW following damage (Mizutari et al., 2011; Stevens et al., 2014; Li et al., 2017).

### Macrophage Depletion Impairs Auditory Function of Postnatal and Young Adult Mice

**Figure 10:**
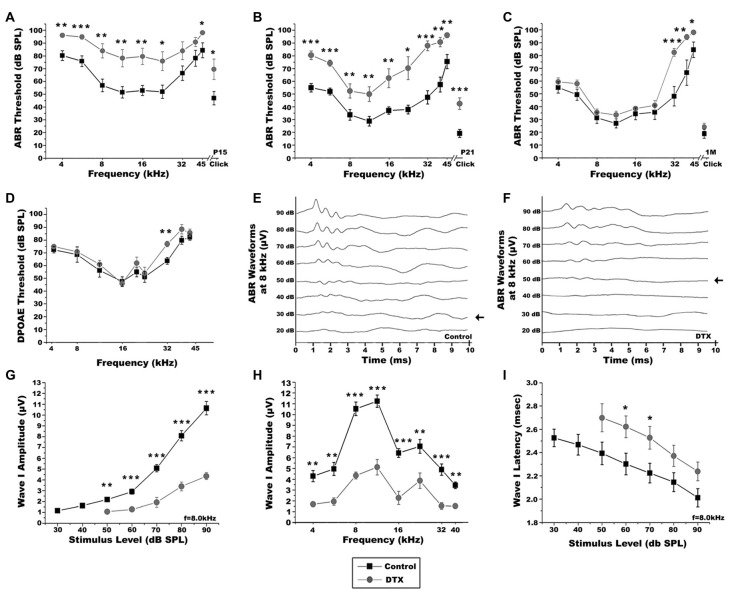
Macrophage depletion impairs auditory function in DT-treated CD11b^DTR/EGFP^ mice. **(A–C)** Auditory brainstem response (ABR) thresholds from P15 **(A)**, P21 **(B)** and 1 month **(C)** control (black) and DTX-treated (gray) CD11b^DTR/EGFP^ mice. ABRs were measured from 4.0 kHz to 42.5 kHz with sound pressure levels from 10 dB to 90 dB. Two-tailed, unpaired Student’s *t*-tests were used to compare thresholds for each group at each frequency (*p < 0.05; **p < 0.01; ***p < 0.001; *n* = 11–13 ears). Error bars represent SEM. **(D)** Distortion product otoacoustic emission (DPOAE) thresholds of 1 month control and DTX-treated CD11b^DTR/EGFP^ mice. DPOAEs were measured from 5.6 kHz to 42.5 kHz with sound pressure levels from 10 dB to 90 dB. Two-tailed, unpaired Student’s *t*-tests were used to compare thresholds for each group at each frequency (**p < 0.01; *n* = 8–10 ears). Error bars represent SEM. **(E,F)** Representative ABR recordings from P21 control **(C)** and DTX-treated **(D)** CD11b^DTR/EGFP^ mice with 8 kHz pure tone stimulus. Arrows indicate the lowest ABR thresholds at this frequency. **(G)** Average wave I amplitudes for P21 DTX-treated (gray, *n* = 4–7 ears) and control (black, *n* = 7–11 ears) CD11b^DTR/EGFP^ mice in response to 8.0 kHz stimulus. DTX-treated CD11b^DTR/EGFP^ mice displayed decreased wave I amplitude responses at 90, 80, 70, 60 and 50 dB SPL. Unpaired Student’s *t*-test analyses were used to compare amplitudes for each group at each stimulus level tested (**p < 0.01; **p < 0.001). Error bars represent SEM. **(H)** Average wave I amplitudes at 4.0–40 kHz for stimuli presented at 90 dB SPL for P21 control and DT treated (gray, *n* = 4–7 ears) and control (black, *n* = 11 ears) CD11b^DTR/EGFP^ mice. Responses are decreased in DTX-treated CD11b^DTR/EGFP^ mice at all frequencies in comparison to control mice. Unpaired *t*-test analyses were used to compare thresholds for each group at each frequency (**p < 0.01; ***p < 0.001). Error bars represent SEM. **(I)** Average Wave I latencies for P21DTX-treated (gray, *n* = 3–8 ears) and control (black, *n* = 7–11 ears) CD11b^DTR/EGFP^ mice in response to 8.0 kHz stimulus. DTX-treated CD11b^DTR/EGFP^ mice displayed longer latency responses at 90, 80, 70, 60 and 50 dB SPL. Unpaired Student’s *t*-test analyses were used to compare amplitudes for each group at each stimulus level tested (*p < 0.05). Error bars represent SEM.

We next investigated how macrophage depletion affected auditory function. ABR tests were performed at P15, after the onset of hearing, and at P21 and 1 month, when auditory nerve function is more mature. At P15, ABR thresholds of DTX-treated mice were higher than controls (Figure 10A). Although ABR responses improved by P21, DTX-treated mice continued to display elevated thresholds when compared to control mice (Figure 10B). Representative ABR waveforms produced in response to tone bursts at 8 kHz in P21 control mice demonstrate characteristic waveforms, similar to those produced by adult mice, while DTX-treated mice produced dampened and wider peaks (Figures 10E,F). By 1 month DTX-treated and control mice demonstrated similar ABR thresholds, with the exception of thresholds produced at the high frequencies (Figure 10C). To determine if OHC loss or dysfunction was present in DTX-treated mice we measured the distortion product otoacoustic emissions (DPOAEs) in the adult mice (Figure 10D). Nearly all frequencies tested, except a slight increase at 32 kHz, showed no difference in DPOAE thresholds when comparing DTX-treated and control responses (Figure 10D).

ABR wave I amplitude input/output function measurements allow the examination of suprathreshold activities of the auditory nerve. ABR wave I latencies give insight into functional integrity of the myelin in the auditory nerve. In DTX-treated mice, ABR wave I amplitudes measured at 8 kHz were decreased compared with controls when presented with stimuli at 90–50 dB SPL (Figure 10G). Wave I amplitudes give insight into the firing synchrony of the auditory nerve. Wave I amplitudes in response to 90 dB stimuli, measured at 4–40 kHz, were significantly lower in DTX-treated mice compared to vehicle-treated mice (Figure 10H). Additionally, increased wave I latencies were observed in DTX-treated mice at 8 kHz when 60–70 dB SPL stimuli were given (Figure 10I). No significant difference was seen in Wave I amplitudes or latencies measured at 8 kHz of 1 month DTX-treated and control mice when presented with stimuli at 90–40 dB (data not shown). These results demonstrate that transient macrophage-depletion impairs auditory nerve function. However, these impairments are temporary and hearing function between DTX-treated and control mice become comparable.

## Discussion

The refinement of auditory nerve fibers during development has been extensively studied. However, it remains unclear how glial cells, which ensheath SGNs and peripheral axons, are affected during the refinement period. In this study, we identify the temporal distribution patterns of cochlear glial cells during auditory nerve refinement. We demonstrate that cochlear macrophages eliminate supernumerary glial cells in the developing auditory nerve. We also show that macrophage depletion in the auditory nerve results in elevated glial cell numbers, abnormal myelin sheath formation, and diminished hearing function. These data reveal a critical role that cochlear macrophages play in glial cell regulation and auditory nerve maturation and hearing onset.

Using immunohistochemical analyses, we found that postnatal glial cells undergo an increase in cell numbers between P3 and P7. Glial cell numbers significantly decrease after the first postnatal week, when mature auditory nerve circuitry is established. It is probable that extra glial cells, termed supernumerary glia in the context of this report, are temporarily produced to support processes important for proper nerve development. Glial cells, such as astrocytes and oligodendrocytes produce neurotrophic factors that are critical for the survival of neurons in the central nervous system (Purves et al., 1988; Zhu et al., 2008). Glial cells from both the central and peripheral auditory nerve promote neuron survival and extension of SGN neurites *in vitro* (Hansen et al., 2001; Jeon et al., 2011). It is possible that supernumerary glial cells are produced to ensure abundant production of trophic factors for the survival of developing neurons. Since glial cells have also been implicated as active participants in nerve refinement, it is also possible that supernumerary glial cells facilitate axonal and synaptic elimination during the auditory nerve refinement process. In mammalian neuromuscular junctions, glial cells facilitate synapse elimination and clear axonal debris during injury-induced nerve retraction (Bishop et al., 2004; Kang et al., 2014; Lee et al., 2016). Further studies are required to understand fully the function of supernumerary glial cells in auditory nerve refinement. It will be important to determine why glial cell numbers decrease following refinement and nerve maturation.

Myelin production and integrity are essential for the proper function and survival of neurons. Complete myelination of SGNs and nerve fibers by glial cells is important for proper auditory signal conduction from the peripheral auditory nerve to central auditory processes (Kim et al., 2013). Mouse models with abnormal myelination in the inner ear, including Connexin29-deficient mice and Saposin B-deficient mice, have been previously described (Tang et al., 2006; Akil et al., 2015). In the inner ear, the gap junction protein Connexin29 is highly expressed in myelinating glial cells that ensheath the somas and processes of SGNs. Connexin29-deficient mice demonstrate abnormal myelination of SGN somas, elevated ABR thresholds, and significantly delayed ABR wave I latencies (Tang et al., 2006). Saposin B is a glycoprotein responsible for lipid transfer in myelin. Satellite cells in the auditory nerve of Saposin B-deficient mice display abnormal inclusion bodies in their myelin sheaths, leading to SGN degeneration and progressive nerve fiber loss (Akil et al., 2015). These pathological alterations result in increased ABR thresholds and delayed wave I latencies (Akil et al., 2015).

Our transcriptomic analysis detected increased expression of immune response genes during the period of nerve refinement, including H2-K1, H2-D1, C1q and C3aR. These results are consistent with a previous study (Lu et al., 2011), suggesting that immune signals play a role in auditory nerve refinement. Previous studies also have identified a role for complement and MHC class I signaling molecules in regulating microglia-mediated nerve pruning in the developing mammalian hippocampus, cerebellum, motor cortex, and optic nerve (Stevens et al., 2007; Paolicelli et al., 2011; Berg et al., 2012; Schafer et al., 2012; Bialas and Stevens, 2013; Dixon-Salazar et al., 2014). Interestingly, C1q deficient and H2-K/H2-D1 double knockout mice do not exhibit altered auditory nerve fiber or synapse numbers (Calton et al., 2014). However, it remains to be determined whether these signaling molecules are involved in other components of auditory nerve development, such as glial cell maturation. It is possible that these molecules could play a role in glial cell maintenance. H2-K/H2-D1 double knockout mice demonstrate progressive hearing impairment, shown by elevated ABR thresholds, suggesting that these mice may possess morphologic abnormalities in the auditory nerve that remain to be identified (Calton et al., 2014). Further exploration of the differentially expressed genes identified in our study may provide insight into molecular cues or gene networks that regulate auditory nerve maturation.

We found that macrophages engulf nerve fiber fragments during auditory nerve refinement. At the same time, macrophages seem to engulf glial cells. Similarly, several studies have shown that microglia or macrophages in the CNS regulate the number of synaptic outputs during development, as well as in adulthood, by engulfing synaptic elements that are misconnected or redundant (Paolicelli et al., 2011; Schafer et al., 2012; Zusso et al., 2012; Chen et al., 2014; Shigemoto-Mogami et al., 2014). Our loss-of-function experiments found that activated macrophages are needed to eliminate supernumerary glial cells during refinement. Quantitative assessment of glial cells remaining in macrophage-depleted auditory nerves revealed a significant increase in glial cell numbers, when compared to controls.

It remains to be determined why supernumerary glial cells are targeted by macrophages for elimination. Our analysis of the ultrastructure in P7 CBA mice revealed that macrophages engulf both myelinating and non-myelinating glial cells as they enwrap degenerating axons. However, it is unknown how targeted glial cells are marked for macrophage phagocytosis. Recent studies have identified several signals that act as “find me” and “eat me” signals for macrophage phagocytosis of apoptotic cells (Truman et al., 2008; Elliott et al., 2009). Identification of glial-related “eat me” signals will be important for further understanding the glia-macrophage relationship we have identified in the developing auditory nerve.

Here, CD11b^DTR/EGFP^ mice treated with DTX at P4/5 demonstrated myelin abnormalities and functional impairments similar to the Connexin29 and Saposin B mouse models mentioned above. DTX-treated CD11b^DTR/EGFP^ mice presented with abnormally high glial cell numbers, the presence of myelin blebbing, abnormal myelination at the paranodes of the nodes of Ranvier, and supernumerary glial cell clustering around SGN axons. However, DTX-treated mice did not demonstrate alterations in neuron numbers or HC innervation and afferent synapse patterns. Our ultrastructural investigation of auditory nerves following macrophage-depletion confirmed the presence of supernumerary glial cells not directly enclosing SGNs or axons. In addition, pathological alterations of myelin sheaths in the auditory nerve suggest that macrophage depletion leads to abnormal glial cell function. Moreover, cochleae of DTX-treated CD11b^DTR/EGFP^ mice exhibited impaired suprathreshold function of the auditory nerve, indicating that proper macrophage activities are critical for auditory nerve maturation and normal hearing function. Further studies will be necessary to determine how elevation of glial cell numbers following macrophage depletion contributes to the pathophysiological changes in the auditory nerve.

DTX-treated mice demonstrated auditory impairment at P15, after hearing function begins. DTX-treated mice continued to produce worsened ABR responses than control mice at P21. Additionally, DTX-treated mice demonstrated dampened wave I amplitudes and increased wave I latencies while DPOAE threshold responses remained comparable between DTX-treated mice and controls. By 1 month of age, control and DTX-treated animals presented similar ABR thresholds, with significant differences only seen in the higher frequencies tested, suggesting that DTX-treated mice eventually employed compensatory mechanisms for auditory nerve maturation.

The stria vascularis maintains the homeostasis of the endolymph in the inner ear. Maintenance of the stria vascularis is essential for the production of the endocochlear potential, which is required for hearing function (Salt et al., 1987; Takeuchi et al., 2000; Juhn et al., 2001). Within the stria vascularis are resident macrophages referred to as perivascular-resident macrophage-like melanocytes. These cells are positive for macrophage-specific markers including F4/80, CD68 and CD11b (Zhang et al., 2012). Previous studies demonstrated that these macrophage-like cells contribute to the integrity of the stria vascularis in adult mice (Zhang et al., 2012). Additionally, disruption of CD11b^+^ cells in the cochlear LW causes leakage from capillaries of the stria compartment, leading to a significant reduction in endocochlear potential and auditory function decline (Zhang et al., 2012, 2013). In the current study, strial edema was seen in the LWs of P11 DTX-treated mice, suggesting that the perivascular-resident macrophage-like melanocytes may be affected by DTX treatment. However, edema was not found in the stria compartment of P16 DTX-treated mice, suggesting that the LW is capable of recovering from macrophage depletion. Since proper generation of endocochlear potential is critical for maintenance of auditory function, it is possible that the changes of LW function also contribute to the temporary hearing impairments seen in the DTX-treated mice. It will be important for future studies to investigate the role of macrophages in LW development and contributions to endocochlear potential of postnatal mice.

Recently, our studies demonstrated that glial cell numbers are increased after auditory nerve injury and that auditory glial cells have the potential to differentiate into neural progenitor cells following acute nerve injury (Lang et al., 2011, 2015). Interestingly, macrophage numbers are increased following auditory injury and are required for SGN protection and the maintenance of SGN numbers after cochlear injury in the adult cochlea (Hirose et al., 2005; Lang et al., 2006, 2016; Okano et al., 2008; Sato et al., 2010; Kaur et al., 2015). Investigation of glial cell refinement and glia-macrophage relationships during development will lead to better understanding of the auditory nerve microenvironment. This will be vital for identifying novel strategies that may promote auditory nerve repair or regeneration.

## Author Contributions

LNB, YX, JLB and HL designed the research; LNB, YX, KVN, JZ, NMS, and JLB performed the research; LNB, YX, KVN, JLB, CHP and MCB analyzed the data; LNB, JLB and HL wrote the article.

## Conflict of Interest Statement

The authors declare that the research was conducted in the absence of any commercial or financial relationships that could be construed as a potential conflict of interest.
